# Prediction and detection of side effects severity following COVID-19 and influenza vaccinations: utilizing smartwatches and smartphones

**DOI:** 10.1038/s41598-024-56561-w

**Published:** 2024-03-12

**Authors:** Yosi Levi, Margaret L. Brandeau, Erez Shmueli, Dan Yamin

**Affiliations:** 1 Department of Industrial Engineering, Tel-Aviv University, Tel-Aviv, Israel; 2 Department of Management Science and Engineering, Stanford University, Stanford, CA USA; 3 MIT Media Lab, Cambridge, MA USA; 4 Center for Combatting Pandemics, Tel-Aviv University, Tel-Aviv, Israel

**Keywords:** COVID-19 vaccine, BNT162b2, Side effects, Vaccine safety, Wearable sensors, Smartwatches, Influenza vaccine, Seasonal influenza, Machine learning, Infectious diseases, Statistics

## Abstract

Vaccines stand out as one of the most effective tools in our arsenal for reducing morbidity and mortality. Nonetheless, public hesitancy towards vaccination often stems from concerns about potential side effects, which can vary from person to person. As of now, there are no automated systems available to proactively warn against potential side effects or gauge their severity following vaccination. We have developed machine learning (ML) models designed to predict and detect the severity of post-vaccination side effects. Our study involved 2111 participants who had received at least one dose of either a COVID-19 or influenza vaccine. Each participant was equipped with a Garmin Vivosmart 4 smartwatch and was required to complete a daily self-reported questionnaire regarding local and systemic reactions through a dedicated mobile application. Our XGBoost models yielded an area under the receiver operating characteristic curve (AUROC) of 0.69 and 0.74 in predicting and detecting moderate to severe side effects, respectively. These predictions were primarily based on variables such as vaccine type (influenza vs. COVID-19), the individual's history of side effects from previous vaccines, and specific data collected from the smartwatches prior to vaccine administration, including resting heart rate, heart rate, and heart rate variability. In conclusion, our findings suggest that wearable devices can provide an objective and continuous method for predicting and monitoring moderate to severe vaccine side effects. This technology has the potential to improve clinical trials by automating the classification of vaccine severity.

## Introduction

Respiratory infectious diseases and their complications have a long history of causing substantial morbidity, mortality, and economic losses. The coronavirus disease 2019 (COVID-19) pandemic had a considerable impact on health and the economy worldwide^1–3^. Furthermore, the World Health Organization (WHO) estimates that about 1 billion cases of seasonal influenza occur each year, of which approximately 3–5 million are severe cases resulting in up to 650,000 deaths^4^. An important tool in the fight against infectious diseases is vaccines^5^. Vaccines aid in the prevention of disease from irremediable conditions by stimulating the body’s adaptive immunity^6^.

Although vaccination is important to lower viral spread, some individuals are hesitant to follow the recommended vaccination advice^7,8^. An important reason for vaccine hesitancy is concern about vaccine safety, efficacy, and side effects^9,10^. COVID-19 vaccines, for example, have been shown to cause numerous adverse side effects ranging from local side effects, such as headache and fever^11,12^ to rare systemic and severe outcomes, such as thrombotic events and myocarditis^11–13^. Anticipating and predicting the adverse side effects of vaccines could help better plan work and leisure schedules a few days after vaccination and increase the vaccination acceptance rate^14^.

Raising confidence in vaccines requires closing the knowledge gap on vaccine safety. The need to rectify this situation is highlighted by the work of the Global Vaccine Data Network. This large vaccine safety project includes scientists from over 20 countries who gather the data needed to investigate rare complications that are linked to COVID-19 vaccines to improve the prediction and treatment of, and potentially prevent, these side effects^15^. While severe events after vaccination likely lead to a medical consultation, which would be noted in the patient’s medical records, milder reactions are generally underreported. To address this issue and provide a comprehensive response to the milder side effects following vaccinations, continuous and detailed detection and surveillance of the side effects following vaccination is needed.

Recent studies have shown a clear association between individual medical and personal characteristics and adverse side effects following COVID-19 vaccinations^12,16^. One study found that sociodemographic variables are potential predictors of differential influenza vaccine outcomes^17^. Besides sociodemographic and self-reported information, an additional source of information on vaccine side effects is data from wearable devices, such as smartwatches. The recent popularity and widespread availability of smartwatch devices enable the collection of huge amounts of data on physiological measures such as an individual’s heart rate, resting heart rate, heart rate variability (HRV), oxygen saturation, and physical activity^18^. Recent studies have used physiological information obtained from wearable devices to prospectively and retrospectively predict and detect COVID-19 infection^19–23^, as well as to examine the duration and variation of recovery among COVID-19–positive vs COVID-19–negative individuals^24^. Other studies using wearables found considerable and short-term changes in heart rate and HRV following vaccination^13,25–28^. However, the identification of vaccine-associated side effects using smartwatch measurements has yet to be established.

This study aims to predict and detect the severity of post-vaccination side effects following BNT162b2 COVID-19 or seasonal influenza vaccination. We designed ML models that utilized sociodemographic, self-reported questionnaires, and smartwatch data as layers of information. Our prediction model can serve as a tool for individuals in anticipating their side effect severity following vaccination, and hence generate a personalized side effects profile, which could potentially increase the vaccination acceptance rate. Likewise, our detection model can potentially allow the classification of vaccine side effect severity in clinical trials in an automated manner.

## Results

### Cohort characteristics

2111 participants received at least one dose of COVID-19 and/or influenza vaccine; 1932 participants received at least 1 dose of BNT162b2 mRNA COVID-19 vaccine between 14 December 2021 and 29 September 2022, and 856 participants received at least 1 dose of seasonal influenza vaccine for the 2021–2022 and 2022–2023 seasons. Of the 2111 participants, 1072 (50.78%) were women and 1038 (49.17%) were men. Their age ranged from 19 to 87 years, with a median age of 52 years (Table 1). A total of 1643 (77.83%) participants had a body mass index < 30 kg/m^2^, and 868 (41.11%) had more than one underlying medical condition (Table 1).

**Table 1:** Information on cohort participants after COVID-19 and influenza vaccination stratified by the presence of self-reported reaction.

	COVID-19 vaccine	Influenza vaccine	COVID-19 and/or influenza vaccine
Total (%)	1932 (100%)	856 (100%)	2111 (100%)
Sex			
Female (%)	979 (50.67%)	442 (51.63%)	1072 (50.78%)
Male (%)	952 (49.27%)	414 (48.37%)	1038 (49.17%)
Not reported	1 (0.06%)	0 (0.00%)	1 (0.05%)
Age (years)			
Mean	49.67	53.59	49.23
Std	15.66	15.23	15.58
Range	19–87	21–85	19–87
Median	53	56	52
Age range			
18–55 (%)	1104 (57.14%)	416 (48.59%)	1249 (59.16%)
> 55 (%)	828 (42.86%)	440 (51.41%)	862 (40.84%)
Underlying medical condition			
Yes (%)	791 (40.94%)	435 (50.81%)	868 (41.11%)
No (%)	1095 (56.67%)	394 (46.03%)	1194 (56.56%)
Not reported	46 (2.39%)	27 (3.16%)	49 (2.33%)
BMI			
> = 30	434 (22.46%)	203 (23.71%)	461 (21.83%)
< 30	1491 (77.17%)	650 (75.93%)	1643 (77.83%)
Not reported	7 (0.37%)	3 (0.36%)	7 (0.34%)

Of the participants who received at least one dose of COVID-19 and/or influenza vaccine and were classified as having “Moderate to severe reaction”, participants > 55 years of age reported fewer reactions than did participants 18–55 years of age, 8.16% vs. 14.72%, odds ratio [OR] 1.94 [95% CI 1.56–2.40, p < 0.0001] (Fig. S1A). Females reported greater rates of “Moderate to severe reaction” than males, 13.07% vs. 9.52, OR 1.42 [95% CI 1.15–1.76, p < 0.001] (Fig. S1B). Participants who had an underlying medical condition reported fewer rates of ”Moderate to severe reaction”, 9.23% vs. 13.17%, OR 1.49 [95% CI 1.20–1.85, p < 0.001] compared with participants who did not have an underlying medical condition (Fig. S1C).

### Descriptive self-reporting patterns

**Figure 1:**
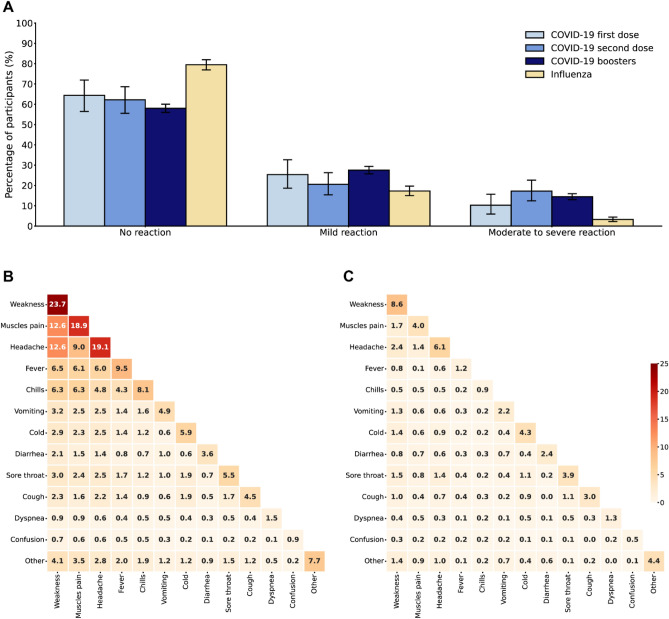
Self-reported reactions to the COVID-19 vaccine compared with the influenza vaccine. (**A**) Percentage of all participants classified into each severity tier based on their most severe reported symptom in the 7 days following vaccination, stratified by vaccine type. Error bars represent 95% CIs. (**B**) Heatmap showing the proportion of post-vaccination self-reported side effects out of all self-reported reactions for participants who received at least 1 dose of the COVID-19 vaccine. Diagonal cells represent individual side effects and the remainder of cells represent sets of side effects. (**C**) Heatmap showing the proportion of post-vaccination self-reported side effects out of all self-reported reactions for participants who received at least 1 dose of the influenza vaccine. Diagonal cells represent individual side effects and the remainder of cells represent sets of side effects.

We classified post-vaccination side effects following COVID-19 and influenza vaccines into “No reaction”, “Mild reaction” and “Moderate to severe reaction” groups, and examined the proportion of participants with “No reaction”, “Mild reaction” and “Moderate to severe reaction” groups following COVID-19 and influenza vaccines for participants who received at least 1 dose of COVID-19 vaccine, and participants who received at least 1 dose of seasonal influenza vaccine. The fraction of participants who received at least one dose of the COVID-19 vaccine and were classified as having “No reaction” was smaller than the fraction of participants who received at least one dose of the influenza vaccine and reported “No reaction”. Specifically, 64.38% (95% CI 56.46–71.92%) of the participants did not report any new symptoms after receiving the first COVID-19 dose, 62.20% (95% CI 55.53–68.64%) after the second COVID-19 dose, and 58.02% (95% CI 55.97–60.06%) after the COVID-19 boosters doses compared with 79.47% (95% CI 76.89–81.93%) after the influenza vaccines (Fig. 1A). Additionally, the proportion of participants who experienced “Mild” or “Moderate to severe” reactions was higher following COVID-19 vaccination compared to the influenza vaccination.

We examined the proportion of individual post-vaccination self-reported side effects out of all self-reported reactions (diagonal cells) and between sets of side effects for participants who received at least 1 dose of the COVID-19 vaccine, and participants who received at least 1 dose of the seasonal influenza vaccine. The most common individual reactions out of all the self-reported reactions for the COVID-19 vaccine were “Weakness” (23.7% of vaccinated individuals), “Headache” (19.1%), “Muscle pain” (18.9%), “Fever” (9.5%), and “Chills” (8.1%) (Fig. 1B). The most common individual reactions out of all the self-reported reactions for the influenza vaccine were “Weakness” (8.6%), “Headache” (6.1%), “Other” (4.4%), “Cold” (4.3%), and “Muscle pain” (4.0%). Notably, the proportion of all individual post-vaccination self-reported side effects was higher for the COVID-19 vaccine than for the influenza vaccine (Fig. 1C) as well as the proportion of simultaneously occurring sets of side effects.

### Descriptive heart measure from smartwatches

**Figure 2:**
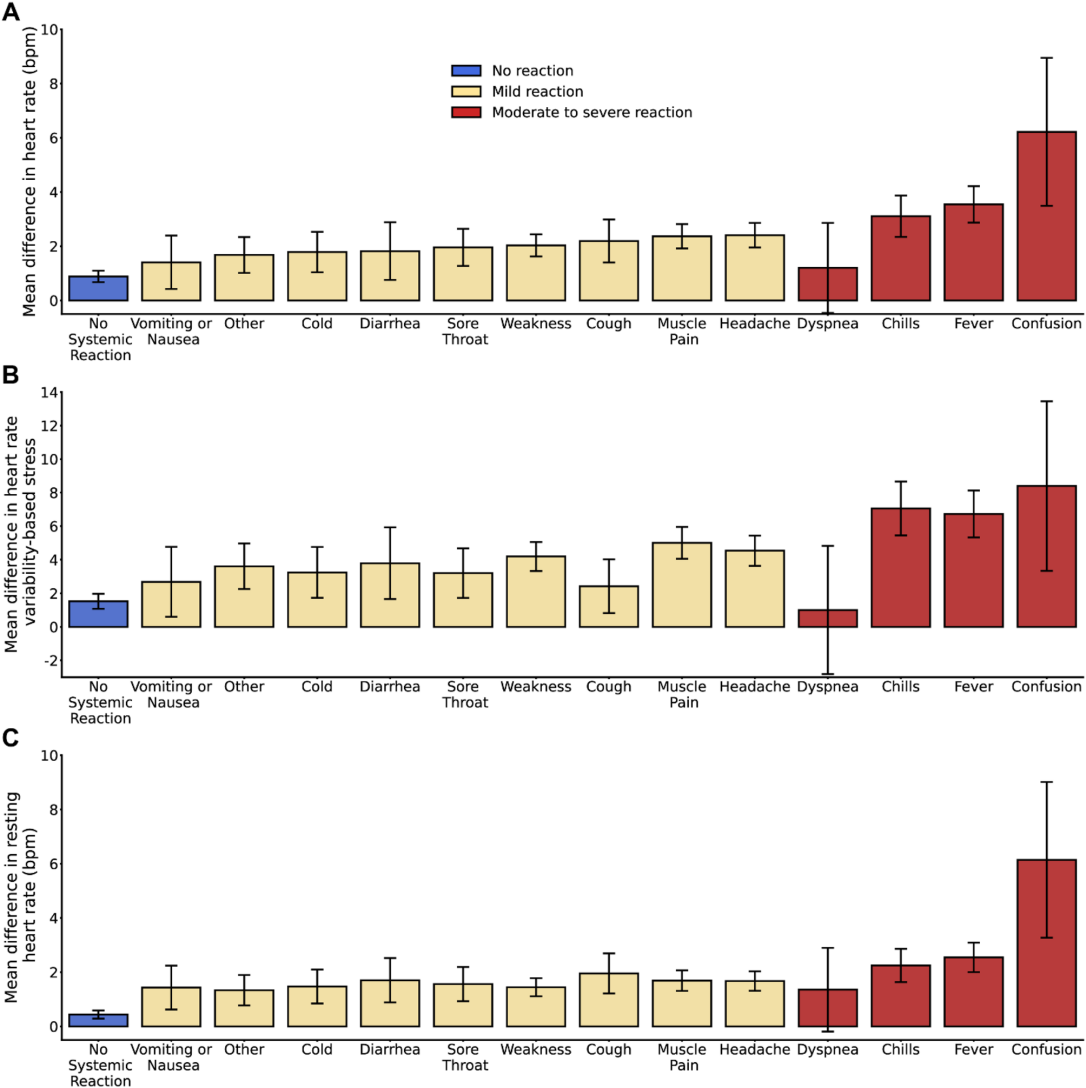
Change in objective and continuous physiologic measurements collected by the smartwatch following COVID-19 and/or influenza vaccine as a function of self-reported side effects. The mean difference in (**A**) heart rate (in beats per minute), (**B**) HRV-based stress (in points), and (**C**) resting heart rate (in beats per minute) between the post-vaccination and baseline periods in Garmin smartwatch data in the 72 h following vaccination. Data information: error bars represent 95% CIs. For each panel, the sample size represents the number of participants for which we had sufficient data points to conduct the analysis, using the criteria presented in the “Materials and methods” section.

In addition to the self-reported data collected from the questionnaires, we analyzed objective and continuous physiologic measurements collected by the smartwatches. We examined the average of the first 72 h post-vaccination for the mean differences between pre-and post-vaccination in hourly heart rate, HRV-based stress, and the first 3 days post-vaccination for the mean differences in daily resting heart rate data for participants who received at least one dose of COVID-19 and/or influenza vaccine (Fig. 2). Participants who reported side effects associated with “Moderate to severe reaction”, except for “Dyspnea”, showed a large increase in the mean difference in heart rate, (Fig. 2A), HRV-based stress levels (Fig. 2B), and daily resting heart rate (Fig. 2C) compared to their baseline levels. These changes were also observed for participants who reported side effects associated with “Mild reaction”, but were considerably lower. Participants who reported “No systematic reaction” had a slight increase in mean heart rate, HRV-based stress, and daily resting heart rate, compared with baseline levels. By the sixth day post-vaccination, heart rate, HRV-based stress, and resting heart rate levels returned to baseline. The differences in physiological measures captured by the smartwatches reflect their ability to detect the severity of self-reported reactions.

### Machine learning model

Following the analysis of the self-reported questionnaires, sociodemographic (age, gender, and background diseases) data, and smartwatch data, we created machine learning models to predict and detect the participant-reported side effect severity following COVID-19 or influenza vaccinations (“No- or Mild-reaction” or “Moderate to severe reaction”). The prediction model utilized sociodemographic, questionnaire, and smartwatch information, but only before vaccine administration, and the detection model additionally utilized the smartwatch measures 72 h post-vaccination information. Four machine learning methods were evaluated for the prediction and detection problems, which are listed with their hyperparameters and performance report (Table S1). Our models included all 2111 patients who received COVID-19 and/or influenza vaccine, corresponding to 2585 COVID-19 and 984 influenza vaccine doses. Of the 3569 vaccine doses, 405 (11.34%) reported having “Moderate to severe reaction”.

**Figure 3:**
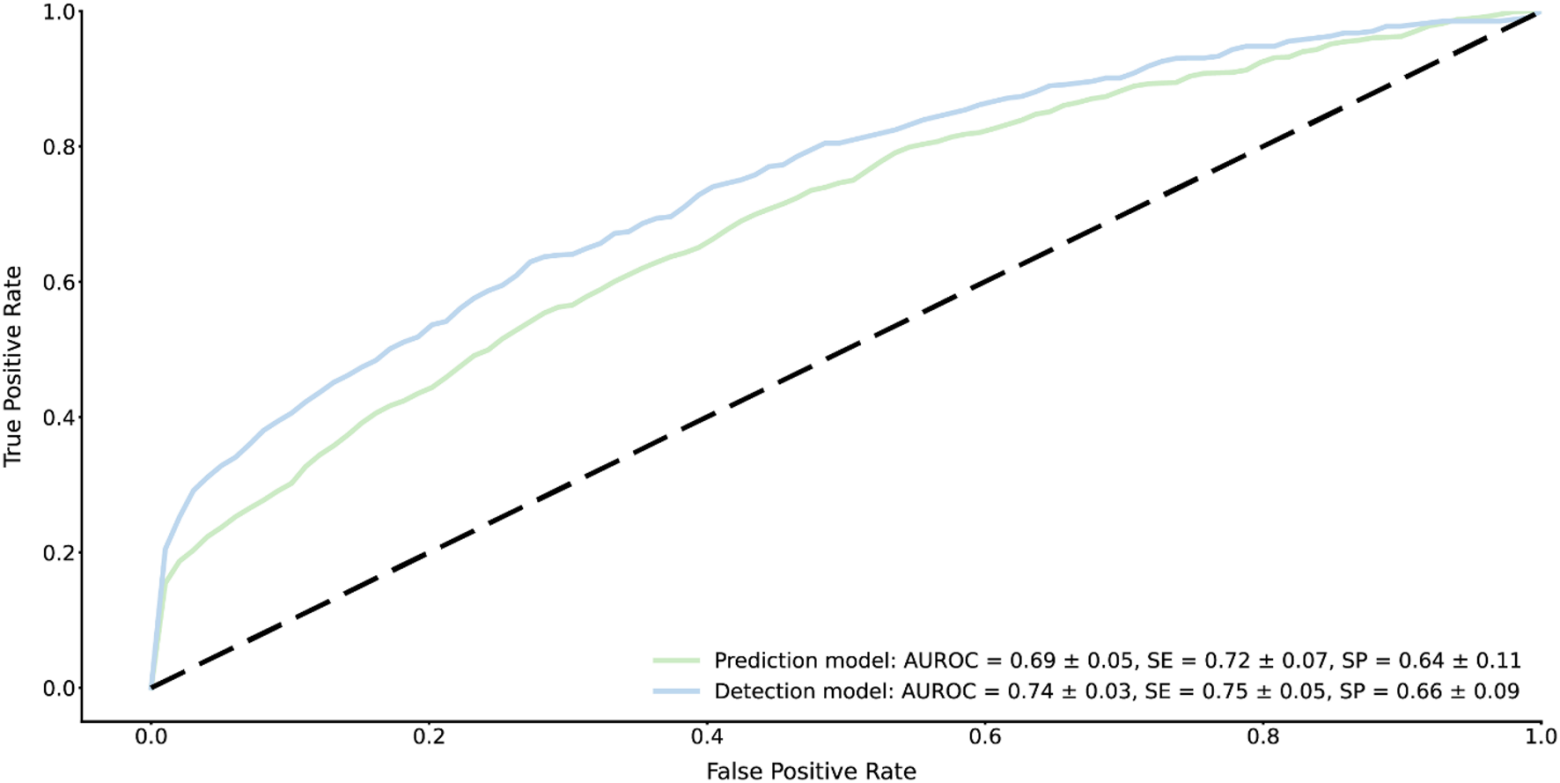
Predictive models’ performance. Receiver operating characteristic curves (ROCs) for a prediction model that utilizes data before vaccination (i.e., sociodemographic, questionnaire, and smartwatch data) (green line), and of a detection model that also utilizes smartwatch data 72 h following vaccination (blue line). Mean values and standard errors for sensitivity (SE), and specificity (SP) are reported, considering the point on the ROC with the highest average value of sensitivity and specificity (Youden index).

XGBoost and RF showed the best performance (Table S1). Notably, the XGBoost model was selected for further analysis. A predictive XGBoost model that combines sociodemographic, questionnaire, and smartwatch layers of information together had a moderate classification ability between “No- or Mild-reaction” and “Moderate to severe reaction”, with an AUROC score of 0.69 ± 0.05 (Fig. 3). A detection XGBoost model that combines that information with the smartwatch measures 72 h post-vaccination had significantly better (p < 0.01) classification ability in participant-reported side effect severity, with an AUROC score of 0.74 ± 0.03 (Fig. 3). This improvement demonstrates the sensitivity of smartwatches to detect the severity of individuals’ side effects from vaccination.

### Model feature importance

**Figure 4:**
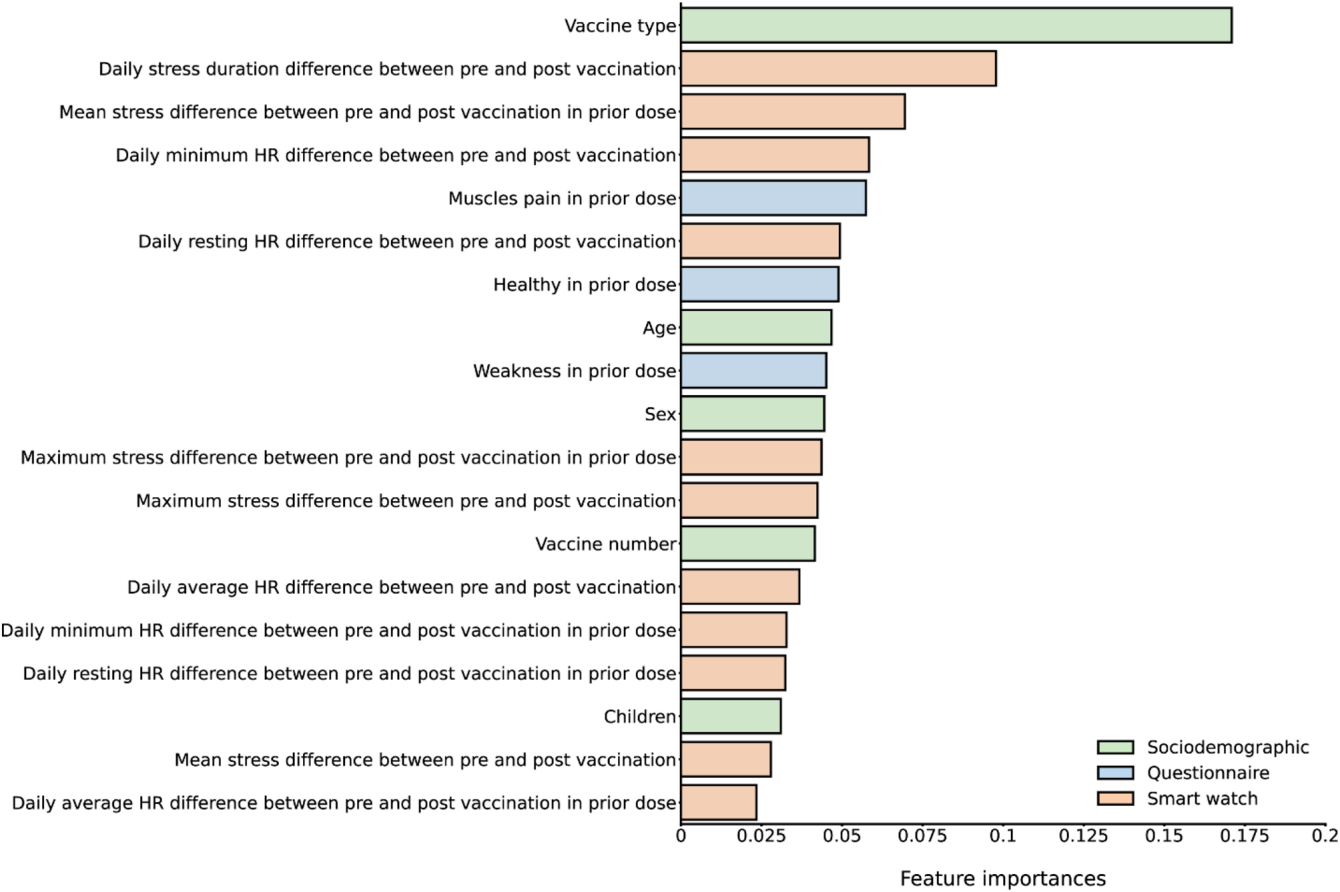
Model feature importance. Contribution of each feature to the detection of the participant-reported side effects following COVID-19 or influenza vaccinations, stratified by sociodemographic, questionnaire, and smartwatch data.

Since the various models used automatic feature selection, delving into the models enabled the assessment of the predictor variable’s importance. The feature importance in the detection model of participant-reported side effects following COVID-19 or influenza vaccinations is shown in Fig. 4. We observed that the highest contribution to the classification ability between “No- or Mild-reaction” and “Moderate to severe reaction” was the vaccine type. This can be attributed to the high percentage of side effects following the COVID-19 vaccine compared to the influenza vaccine. Sociodemographic data had the least contribution to the detection model. Smartwatch data following vaccination, as well as smartwatch data collected in previous doses, had a significant contribution to the classification ability between “No- or Mild-reaction” and “Moderate to severe reaction” in the detection model: for example, an increase in stress duration following vaccination was a classification predictor for the severity of post-vaccination side effects. Likewise, the presence of some side effects (“Muscle pain” and “Weakness”) or the absence of them (“Health”) following a specific dose increased the classification ability after subsequent doses.

## Discussion

In this study, we developed machine-learning-based approaches to predict and detect the severity of post-vaccination side effects following BNT162b2 COVID-19 or seasonal influenza vaccines by utilizing specific data collected from the smartwatches, including resting heart rate, heart rate, and HRV-based stress, combined with sociodemographic data and data from self-reported questionnaires. We found that participants who received at least one dose of COVID-19 and/or influenza vaccine, < 55 years of age, female sex, and participants without an underlying medical condition had greater self-reported local and systemic reactions. This result is consistent with recent studies^16,27^. The proportion of participants who experienced “Moderate to severe reaction” was higher following the COVID-19 vaccination than for the influenza vaccination, for which participants reported mostly “No reaction”. Indeed, it has been shown that COVID-19 vaccination is more closely associated with systematic side effects such as chills, fever, confusion, weakness, and muscle pain, while the influenza vaccine is associated with local injection site side effects^29^.

We found that the elevation in heart rate, HRV-based stress, and resting heart rate measures post-vaccination correlated with the severity of the side effects. Participants reporting “Moderate to severe reaction” had a significant increase in those measures, compared to participants reporting “Mild reaction”. Importantly, even among participants who did not report side effects after vaccination, the smartwatch detected a significant physiological response compared to the baseline period in the first 72 h after vaccination. Furthermore, an ML approach that uses sociodemographic, self-reported questionnaire, and smartwatch data to predict participant-reported side effect severity following COVID-19 or influenza vaccinations (“No- or Mild-reaction” or “Moderate to severe reaction”) had an AUROC of 0.69. A detection model that combines that information with smartwatch measures 72 h post-vaccination had a significantly better performance with an AUROC of 0.74. These results demonstrate the greater sensitivity of wearable devices in detecting side effect severity following COVID-19 or influenza vaccinations. In addition, our results underscore the need for integrated ML models that utilize data from wearables to classify the severity of reactions following vaccines in clinical trials.

Our study has several limitations. First, the participants were slightly older than the Israeli population, so our analyses and ML models might not be generalizable to the entire Israeli or global population. However, the side effects types, frequency, and duration we observed for the COVID-19 and influenza vaccines were similar to those observed in the BNT162b2 mRNA and inactivated influenza vaccines clinical trials^30,31^. Second, all participants received the BNT162b2 mRNA COVID-19 between 14 December 2021 and 29 September 2022 and seasonal influenza vaccines for the 2021–2022 and 2022–2023 seasons. However, studies have shown that different COVID-19 vaccines have similar profiles^31,32^. Likewise, although the influenza vaccine usually changes each year due to the frequent appearance of new strains, the extent and the types of local and systemic reactions are similar. Therefore, we believe that applying our analyses and ML models to other COVID-19 and influenza vaccine types is likely to yield similar results. Third, we assume that participants’ behaviors would be the same throughout the pre-and post-vaccination periods. However, factors that are not associated with vaccination (e.g., caffeine or alcohol consumption) could influence physiological measures in the analysis of the mean difference between the two time periods. Fourth, the smartwatches used in this study are not medical-grade devices, nor are they representative of all wearable devices. Nevertheless, recent studies show that previous versions of such devices have high accuracy for physiological measurements^33,34^. Fifth, participants were not able to report the levels of symptom severity in the questionnaire, and therefore, smartwatch objective physiological measurements could be significantly different between individuals, which may affect the performance of our ML models. Lastly, in our analysis, we focused on a constrained dataset encompassing only the week prior to vaccination and the 72 h following each vaccination event for each participant. We derived a limited set of non-sequential predictive variables and employed off-the-shelf, non-sequential machine learning models, which are not optimized for processing temporal data. We recognize that the model's predictive capabilities could potentially be enhanced by incorporating longer time series data. This could include daily questionnaires capturing symptoms not directly related to vaccines, as well as extended data gathered from smartwatches. Additionally, the use of sequential machine learning models, as demonstrated in a previous study^35^, might further improve the accuracy and relevance of our predictions.

In conclusion, our ML models that combine sociodemographic, self-reported questionnaires, and smartwatch data can predict and detect participant-reported side effect severity following COVID-19 or influenza vaccinations. The ability to predict or detect the participant-reported side effect severity can be improved by including more input and output data. Importantly, this approach can be applied to other vaccines and drugs. The sensitivity of wearable devices allows better detection ability of the side effect severity following COVID-19 or influenza vaccination, and will potentially improve clinical trials by enabling the classification of vaccine severity as well as earlier identification of abnormal reactions in an automated manner.

## Materials and methods

### Study design and participants

We studied a cohort of 2111 participants (> 18 years of age) who were recruited between 14 December 2021 and 29 September 2022 to the PerMed prospective observational study from all across Israel^27,36^. Of the 2,111 participants, 1932 participants reported receipt of > 1 doses of the BNT162b2 mRNA COVID-19 vaccine, and 856 participants reported receipt of > 1 doses of the influenza vaccine. Specifically, of the 1932 participants receiving COVID-19 vaccination, during the study, 146 received their first dose, 209 their second dose, 1545 their third dose, and 685 their fourth dose. Of the 856 participants who received influenza vaccination, 791 received the seasonal dose for 2021–2022, 188 received the seasonal dose for 2022–2023, and 125 received both seasonal doses. Participant recruitment was conducted via advertisements on social media and word-of-mouth. Each participant signed an informed consent form after receiving a comprehensive explanation of the study from a professional survey company. The participants were equipped with a Garmin® Vivosmart 4 smartwatch and installed two apps on their smartphones: a dedicated mobile application (PerMed App), that collected daily self-reported questionnaires^37^, and an application that passively recorded the smartwatch data. Further information regarding data collection architecture and the PerMed dashboard is provided in our previous works^13,25,27^ and the Supplementary Material (Appendix B).

### Participant recruitment and engagement

We hired a professional survey company to recruit participants as well as to keep them engaged throughout the PerMed study. The survey company was responsible for guaranteeing that the participants met the study’s requirements, in particular, that they agreed to wear the smartwatch and fill in the daily questionnaires at least three times a week. We implemented several measures to minimize attrition and churn of participants and consequently improve the quality, continuity, and reliability of the collected data. First, participants who did not fill out the daily questionnaire by 7 p.m. received a notification in their mobile app to fill out the questionnaire. Second, a dedicated dashboard that allowed the survey company to identify participants who continually neglected to complete the daily questionnaires at least three times a week or did not wear their smartwatch for a long duration of time was developed. These participants were contacted by the survey company (either by text messages or phone calls) and were encouraged to better adhere to the study protocol. Third, to strengthen participants’ engagement, a weekly personalized summary report was generated for each participant and was available inside the PerMed application. Similarly, a monthly newsletter with recent findings from the study and useful tips regarding the smartwatch’s capabilities was sent to the participants. At the end of the study, participants will receive all personal insights that were obtained and can keep the smartwatch as a gift.

### PerMed mobile application

After joining the PerMed study, participants filled out the enrollment questionnaire, and information on participants’ sex, age, and underlying medical conditions, was collected. The list of underlying medical conditions consisted of hypertension, diabetes, heart disease, chronic lung disease, immune suppression, cancer, renal failure, and body mass index (BMI) > 30 (BMI is defined as weight in kilograms divided by the square of height in meters). Participants filled out a daily questionnaire through the PerMed mobile application^27,36^. The questionnaire allowed participants to report their signs and symptoms from a closed list of local and systemic reactions previously observed in BNT162b2 mRNA COVID-19 or influenza vaccines^31,38^, with an option to add other symptoms as free text. A detailed description of the questionnaire is provided in the Supplementary Material (Appendix A pp 6–8).

### Smartwatch

Participants were equipped with Garmin® Vivosmart 4 smart fitness trackers. Among many physiological measurements, the smartwatch provides continuous measures of heart rate, stress, and daily resting heart rate capabilities^39^. Since the HRV measure is not accessible through Garmin’s application programming interface, we used Garmin’s stress level measure instead, which is computed based on the HRV measure^40^. HRV-based stress is a measure between 1 to 100 computed by Garmin and is categorized into four tiers: rest (1–25), low (26–50), medium (51–75), and high (76–100)^41^. Specifically, the Garmin device uses heart rate data to determine the interval between each heartbeat. The variable length of time between each heartbeat is regulated by the body’s autonomic nervous system. Less variability between beats correlates with higher stress levels, whereas an increase in variability indicates less stress^41–43^. When we examined data collected in our study, we identified a heart rate sample approximately every 15 s, a stress sample every 180 s, and a daily sampling of resting heart rate.

### Data preprocessing

We performed several preprocessing steps on the daily questionnaire data and smartwatch physiological measures before analyzing the data. For the daily questionnaires, if participants filled in the daily questionnaire more than once on a given day we considered only the last entry reported. For the HRV-based stress and heart rate measures collected by the smartwatches, we computed the mean value for each hour of data. Then, to impute missing values, we performed a linear interpolation. Finally, data was smoothed by calculating the moving average value using a 5-h sliding window.

### Data analysis and inclusion criteria

For each participant, we defined the 7 days before vaccination as a baseline period. For the analysis which involves self-reported questionnaires and for the machine learning model, we included only participants who filled out at least one questionnaire during the baseline period and at least one questionnaire during the 7 days following vaccination. Those questionnaires are required to determine whether symptoms reported by the participants should be considered side effects. We defined a reaction as a post-vaccination side effect if it had not been reported during the baseline period. For the questionnaire data, we calculated the percentage and corresponding 95% CI of participants who reported new systemic reactions in the 7 days following vaccination. We used a Beta distribution to calculate the 95% CI.

For the analysis involving smartwatch measurements, we included participants who had at least one overlapping period of data (i.e., the same day of the week and same hour during the day for the heart rate and HRV-based stress measures, and the same day of the week for the daily resting heart rate) during their baseline and post-vaccination periods. The overlapping periods are required for computing the change in measurement values between the baseline and post-vaccination periods. To calculate the changes in continuous Garmin smartwatch measurements (heart rate and HRV-based stress measures) over the 0–7 days post-vaccination, with those of the baseline period, we calculated for each participant the difference between the measurement of each hour during the seven days post-vaccination and that of the corresponding hour’s in the baseline period (keeping the same day of the week and the same hour during the day). For the daily resting heart rate, we calculated the differences in the same manner (keeping the same day of the week). A Randomized control trial^31^, and prior studies analyzing physiological measures via smartwatches and self-reported questionnaires^13^, demonstrate a significant decrease in local and systemic reactions within 72 h post-vaccination. Consequently, our classification problem focused on determining whether a moderate to severe reaction occurred within this 72 h post-inoculation period.

Based on data from the Centers for Disease Control and Prevention, we stratified the participant-reported post-vaccination side effects by the severity of the reactions they reported in the questionnaire in the post-vaccination period by the appearance of symptoms, as follows:

1. “No reaction”
2. “Mild reaction”: abdominal pain, back or neck pain, feeling cold, muscle pain, weakness, headache, dizziness, vomiting, sore throat, diarrhea, cough, leg pain, ear pain, loss of taste and smell, swelling of the lymph nodes.
3. “Moderate to severe reaction”: fast heartbeat, hypertension, chest pain, dyspnea (shortness of breath), fever, confusion, and chills.

Participants were classified into one of the three categories, based on the most severe symptom that was reported in their post-vaccination period.

### Machine learning model

We stratified participants by the severity of their reactions. Participants who did not report a reaction, or had a “Mild reaction” following vaccination were classified in the “No- or Mild-reaction” group, and the remaining participants were classified in the “Moderate to severe reaction” group. We developed ML models to predict and detect the participant-reported side effect severity following COVID-19 or influenza vaccinations. The prediction model utilized sociodemographics, side effects from previous doses collected from questionnaires, and smartwatch information, but only before the vaccine, while the detection model also utilized the smartwatch measures 72 h post-vaccination information.

The entire data set has been randomly divided into 5 separate non-overlapping test sets. For each test set, a model is trained using all the remaining data, ensuring an equal percentage of positive cases between train and test sets to take into account imbalanced positive and negative classes.

Several machine learning techniques were evaluated for both models: Gradient Boosted Decision Trees (XGBoost), Random Forest (RF), Multi-Layer Perceptron (MLP), and K-Nearest Neighbors (KNN).

The XGBoost package was used for training Gradient Boosted Decision Trees^44^, while the Scikit-learn machine learning library was used to implement the other models^45^.

Performances of the testing samples from each model are reported by mean AUROC, sensitivity (SE), and specificity (SP). SE and SP are defined as the fraction of positive and negative individuals correctly classified, respectively. These values are based on the point in the ROC that optimized the Youden index^46,47^. For each classifier, we applied a grid search within our stratified cross-validation framework and optimized our model selection using the mean AUROC.

The interpretable nature of the decision tree model allows for the evaluation of feature importance estimates^48^. The XGBoost in-model feature importance was used to demonstrate each predictor variable’s effect on the detection of the participant-reported side effect severity.

For evaluation of the differences in terms of AUROC, a bootstrap test (n = 1000) for the difference was used. We repeatedly sampled the dataset with replacement in a stratified manner. We trained the prediction and detection models for each bootstrap sample and computed AUROC on the unique data points that were not selected in the current bootstrap sample. Each model is trained and tested with its subset features and the best hyperparameters. For each bootstrap sample, we computed the AUROC difference between the prediction and detection models and generated a distribution of bootstrapped differences. Finally, we calculated the p-value which is the proportion of bootstrapped differences that is less than or equal to 0.

### Ethical approval

Before participating in the study, all subjects were advised, both orally and in writing, as to the nature of the study and gave written informed consent to the study protocol, which was approved by the Tel Aviv University Institutional Review Board (0002522-1). All methods were performed in accordance with the relevant guidelines and regulations.

### Supplementary Information

Supplementary Information.

## Data Availability

Access to the data used for this study can be made available upon request and is subject to internal review approval from the institutional review board of Maccabi Healthcare Services (MHS) with the current data-sharing guidelines of MHS and Israeli law.
