# Supplementary material for: Prediction and detection of side effects severity following COVID-19 and influenza vaccinations: utilizing smartwatches and smartphones
Source: Sci Rep. 2024 Mar 12;14:6012. doi: 10.1038/s41598-024-56561-w (PMC10933398; doi:10.1038/s41598-024-56561-w)
Supplement: Supplementary file 1 — Supplementary Information. [file 41598_2024_56561_MOESM1_ESM.docx]

**Supplementary Materials for:**

**Prediction and detection of side effects severity following COVID-19 and influenza vaccinations—utilizing smartwatches and smartphones**

**Yosi Levi^1^, Margaret L. Brandeau^2^, Erez Shmueli^1,3^, Dan Yamin^1,2,4,*^**

^1^Department of Industrial Engineering, Tel-Aviv University, Tel-Aviv, Israel

^2^Department of Management Science and Engineering, Stanford University, Stanford, California, United States of America

^3^MIT Media Lab, Cambridge, MA, USA

^4^Center for Combatting Pandemics, Tel-Aviv University, Tel-Aviv, Israel

*****Corresponding author: dan.yamin@gmail.com

Dan Yamin

Department of Industrial Engineering

Tel-Aviv University

Tel-Aviv, Israel

Tel: +972-3-640-7332, Fax: +972-3-640-9160

**Keywords**: COVID-19 vaccine, BNT162b2, side effects, adverse effects, wearable sensors, smartwatches, Influenza vaccine, seasonal Influenza, network analysis

Table of Contents

[**Appendix A – Study protocol** 3](#_Toc156674445)

[Study Design 3](#_Toc156674446)

[Validation of sample size 3](#_Toc156674447)

[Participants 3](#_Toc156674448)

[Study Procedures 4](#_Toc156674449)

[Enrollment Questionnaire 4](#_Toc156674450)

[Monitoring Device 4](#_Toc156674451)

[Vaccination Questionnaire 5](#_Toc156674452)

[Daily Questionnaires 6](#_Toc156674453)

[Data Storage 8](#_Toc156674454)

[Potential Risks & Risk Management 9](#_Toc156674455)

[Privacy/Confidentiality 9](#_Toc156674456)

[**Appendix B – Data collection platform and data access** 10](#_Toc156674457)

[Architecture 10](#_Toc156674458)

[The PerMed Dashboard 11](#_Toc156674459)

[The Type of Data Collected and Data Access 11](#_Toc156674460)

[**References** 12](#_Toc156674461)

[**Supplementary Figures** 13](#_Toc156674462)

[**Supplementary Tables** 16](#_Toc156674463)

**Appendix A – Study protocol**

Study Design

In this study, we will analyze data that were already collected and will be collected as part of the PerMed study^1^. Participants in the PerMed study are recruited for two years, during which they are equipped with a Garmin Vivosmart 4 smartwatch and are asked to wear it as much as they can. In addition, participants installed two applications on their mobile phones: an application that passively collects data from the smartwatch and a dedicated mobile application that allows participants to fill out a daily questionnaire and to report their vaccine date and specific hour. In this study, we will consider for each participant, the 7 days before any vaccination dose as the baseline period.

Validation of sample size

Our dataset included 1,932 participants vaccinated with the COVID-19 vaccine and 856 with the influenza vaccine. To validate the adequacy of these sample sizes, we conducted a thorough review of relevant literature. Studies, encompassing randomized control trials (RCTs) and previous observational cohorts, reveal that 20-40% of individuals experience systemic reactions post-COVID-19 vaccination, and 5-15% post-influenza vaccination^2–4^. Additionally, physiological changes detectable through wearables can occur even in those reporting no symptoms^2^. With an assumption of normal distribution for these proportions and a conservative 2.5% margin of error, the minimum required sample size is 292 for influenza and 984 for COVID-19. Our study comfortably surpasses these minimum thresholds, thereby confirming the robustness of our sample size.

Participants

The inclusion criteria for the PerMed study include those aged > 18 years. Individuals who are not eligible to give and sign a consent form of their free are excluded. In this study, we will analyze the data of participants aged 18 years and above, who reported receiving at least one dose of the BNT162b2 mRNA COVID-19 vaccine or seasonal influenza vaccine after joining the PerMed study. To recruit participants and ensure they complete all the study’s requirements, we will hire a professional survey company. Potential participants will be recruited through advertisements on social media, online banners, and word-of-mouth. The survey company is responsible for guaranteeing the participants meet the study’s requirements, in particular, that the questionnaires are filled daily, ensuring the smartwatches are charged constantly and worn properly, and assisting participants in resolving technical problems.

Study Procedures

Before participation in the study, all participants will be advised orally and in writing about the nature of the experiments and give written informed consent. At this time, participants will be asked to complete an enrollment questionnaire that includes demographic information and health status. In addition, participants will be asked to install two applications on their mobile phones: an application that passively collects data from the smartwatch and the PerMed application, which allows participants to fill in the daily questionnaires. Participants will be given instructions regarding the self-reported symptoms questionnaires and how to operate the smartwatch, which they will wear as much as they can.

Enrollment Questionnaire

All participants will fill out a one-time enrollment questionnaire that includes demographic questions and questions about the participant’s health condition in general. Specifically, the questionnaire will include the following: age, gender, height, weight, and underlying medical conditions (Listed in Table 1, main text). Other questions such as name, address, phone, and email will be recorded and used by the survey company to contact the participants. The answers will be filled in directly by the survey company to the study’s secured dashboard.

Monitoring Device

Participants will be equipped with Garmin Vivosmart 4 smart fitness trackers. Among other features, the smartwatch provides all-day heart rate and heart rate variability and during-night blood oxygen saturation level tracking capabilities^5^.

The optical wrist heart rate (HR) monitor of the smartwatch is designed to continuously monitor a user’s heart rate. The frequency at which heart rate is measured varies and may depend on the level of activity of the user: when the user starts an activity, the optical HR monitor’s measurement frequency increases.

Since heart rate variability (HRV) is not easily accessible through Garmin’s application programming interface (API), we use Garmin’s stress level instead, which is calculated based on HRV. Specifically, the device uses heart rate data to determine the interval between each heartbeat. The variable length of time between each heartbeat is regulated by the body's autonomic nervous system. Less variability between beats correlates with higher stress levels, whereas an increase in variability indicates less stress^6^. A similar relationship between HRV and stress was also seen in^7,8^.

The Pulse Ox monitor of the smartwatch uses a combination of red and infrared lights with sensors on the back of the device to estimate the percentage of oxygenated blood (peripheral oxygen saturation, SpO2%). The Pulse Ox monitor is activated each day at a fixed time for a period of four hours (the default is 2 AM-6 AM).

Examining the data collected in our study, we identified an HR sample roughly every 15 seconds, an HRV sample every 180 seconds, and a SpO2 sample every 60 seconds.

While the Garmin smartwatch provides state-of-the-art wrist monitoring, it is not a medical-grade device, and some readings may be inaccurate under certain circumstances, depending on factors such as the fit of the device and the type and intensity of the activity undertaken by a participant.

Vaccination Questionnaire

The vaccination questionnaire required the following data:


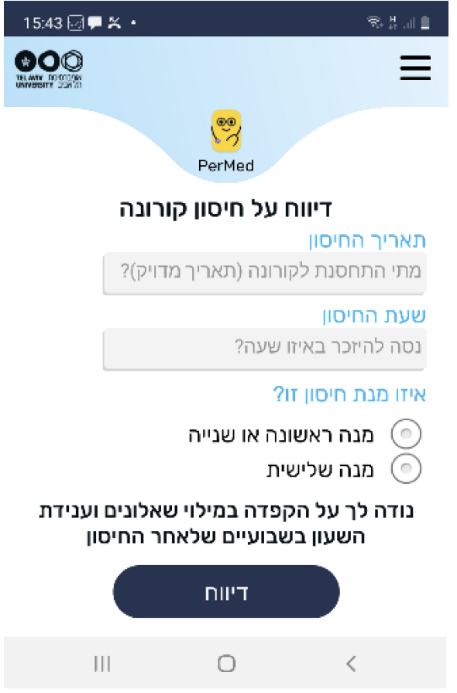
COVID-19 vaccination – date, time, and dose number. [note, this is for validation as vaccination data are reported in the EMR]

Daily Questionnaires

All participants will complete the daily self-reported questionnaire in a dedicated application (the PerMed mobile application). The daily questionnaire we will use includes the following questions:


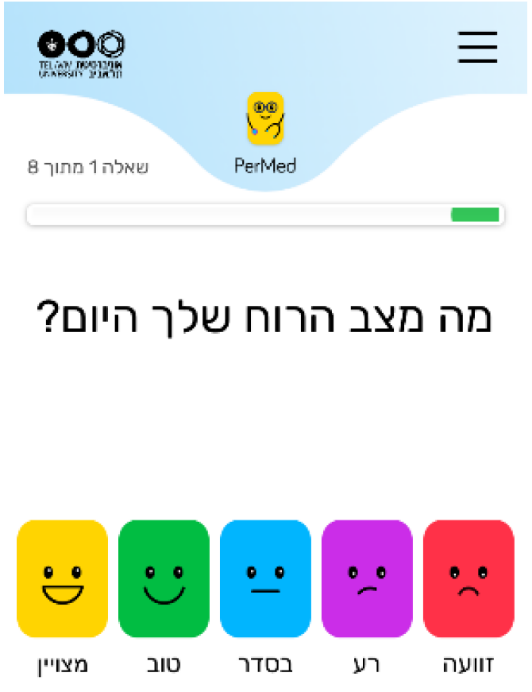
How is your mood today? • Awful (-2)• Bad (-1)• OK (0)• Good (1)• Excellent (2)


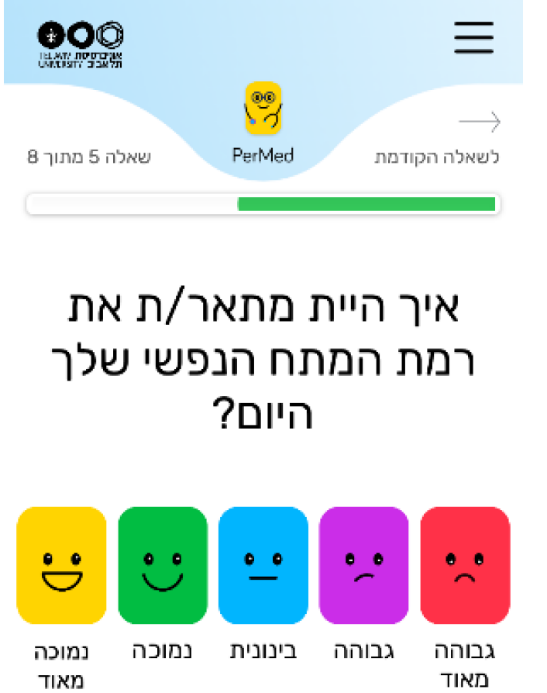
How would you describe the level of your stress during the last day?• Very Low (-2)• Low (-1)• Medium (0)• High (1)• Very high (2)


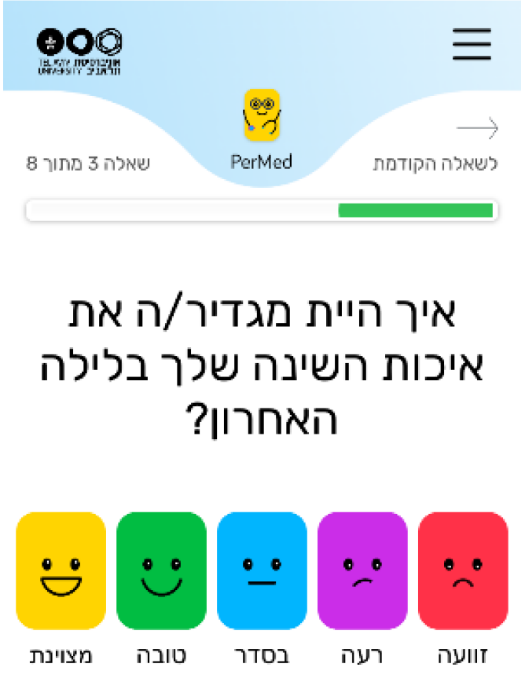
How would you define your last night sleep quality?• Awful (-2)• Bad (-1)• OK (0)• Good (1)• Excellent (2)


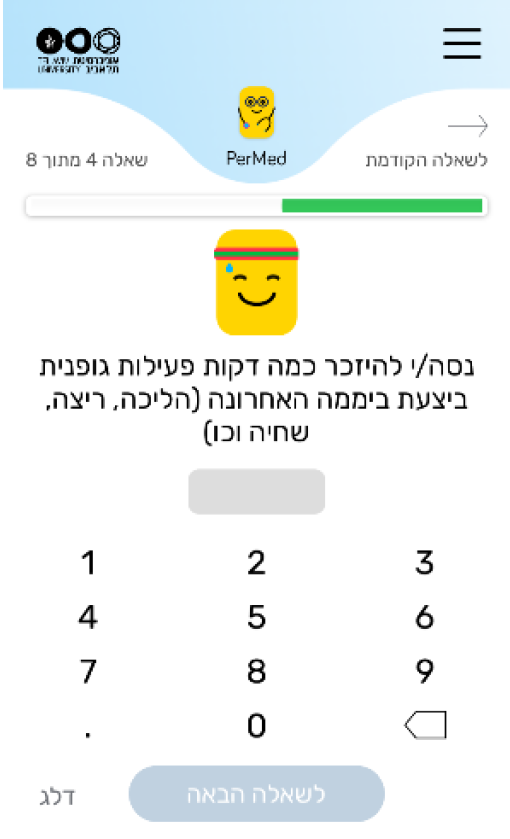
Try to remember how many minutes of sports activity you performed on the last day.


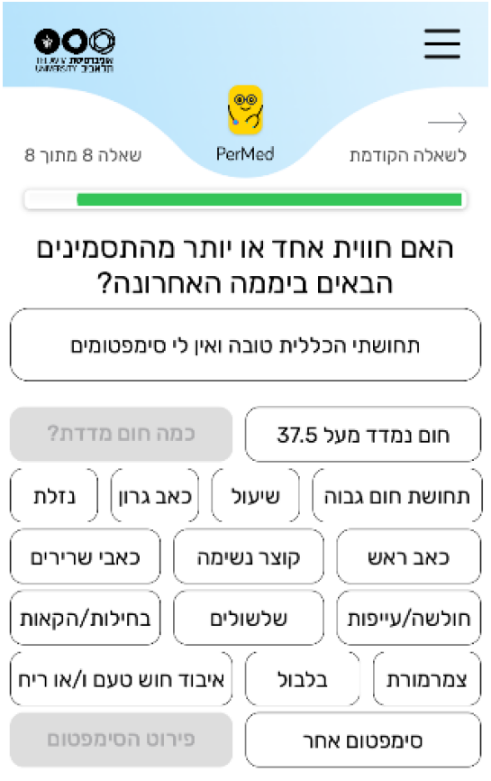
Have you experienced one or more of the following symptoms in the last 24 hours?• My general feeling is good, and I have no symptoms• Heat measured above 37·5• Cough• Sore throat• Runny nose• Headache• Shortness of breath• Muscle aches• Weakness / fatigue• Diarrhea• Nausea / vomiting• Chills• Confusion• Loss of sense of taste / smell• Another symptom.

Data Storage

Data collected from the mobile phone application and the smartwatches will be stored on a secure server within Tel-Aviv University facilities. The server runs a CentOS operating system and is located in the Software Engineering Building at Tel-Aviv University. This server is protected behind the university's firewall and is not connected to external networks. In addition, a secure connection through an SSL protocol and a trusted certificate will be obtained for the transfer of information from the mobile phone application into the secured server.

Access will be restricted to investigators in the study. The information from the mobile application will be stored in a structured manner on the secured server without any explicitly identifying information (name, ID number, email). Each participant will be assigned a coded participant number that will be used to identify the subject in the database. The code with the identified information will be stored in an encrypted form on a separately secured server that only the research manager will have access to. Access to all servers is restricted with a username and password.

All (non-digital) questionnaires and signed informed consent documents will be stored in a secured cabinet at Tel-Aviv University, to which only the research manager and the principal investigators will have access. No data collected as part of the study will be added to individuals’ medical charts.

Potential Risks & Risk Management

No specific risks arising from the smartwatches are expected, as the device is already commercialized with no known adverse reactions. The main risk in this study is the leakage of private data which we intend to manage as we describe in the following section.

Privacy/Confidentiality

Results from this study will be handled at an aggregated level. Individual data records will remain confidential and will not be published or shared with any third party. Signed and dated informed consent forms, as well as data recording sheets (e.g., case report forms) will be stored in locked cabinets during the study and following its completion. A file containing the personal details of the participants will be coded to help preserve confidentiality and will be separated from all other data collected throughout the study. This file will be kept by the principal investigator. Data will be stored on computers in password-protected files.

The data obtained from the smartwatch used in this study will be linked to a coded participant number. The smartwatch does not include a GPS. The data collected by the PerMed application will arrive directly into PerMed back-end servers and will be stored securely.

**Appendix B – Data collection platform and data access**

Architecture

The data collection platform contains several components that interact with each other (Fig. S2):

- **The PerMed application** - This application is installed on each participant’s phone to collect sensor data and the self-reported daily questionnaires. It also handles the smartwatch pairing. The current version of the application supports both Android and iOS devices.
- **The smartwatch** - sends the data to the Garmin Connect app on the smartphone, which then sends these data to Garmin’s server.
- **The smartwatch application** - This application (currently Garmin) receives information from the smartwatch via Bluetooth and transmits it to the company's server. In addition, it provides a convenient interface for displaying the participant's smartwatch information.
- **The app server** - The web server handles the database connectivity using REST API pages. It enables the server to authenticate users as they launch the application and write records to the database. A MySQL server stores the sensors' raw data and the answers to the daily questionnaires. At last, there is a batch process running on the server that sends app notifications (daily reminders to fill out the questionnaire).
- **The dashboard server** - hosts the dashboard pages, which assist in monitoring the quality of the information and controlling the experiment. The dashboard has access to participant information and signals indicating whether questionnaires were completed and the smartwatch was worn without seeing its content directly. A batch process is responsible for aggregating raw data for dashboard statistics.
- **The smartwatch server** - A MySQL server stores the smartwatch data. A batch process is responsible for collecting the data from the Garmin server.

The PerMed Dashboard

Participants will be recruited by a qualified external recruitment team headed by Tel-Aviv University personnel. The team receives limited information essential to control the experiment. Thus, we developed a dedicated dashboard for monitoring the quality of the information and controlling the experiment. This dashboard aims to identify data collection issues such as participants who did not fill out the daily questionnaires or participants who did not charge the battery of their smartwatches. The dashboard also helps us identify problems that were not related to participants’ cooperation, such as bugs in the mobile app. This identification allows us to respond faster and provide timely solutions.

The Type of Data Collected and Data Access

Data collected by the platform arrive from four primary sources:

- **Enrolment questionnaire** - data were collected from a one-time enrollment questionnaire that includes basic personal characteristics such as socio-demographic information (e.g., age, gender, height, weight), general habits, health status, and a short Big Five personality questionnaire.
- **Daily questionnaire** - consists of questions on 1) well-being, 2) general health condition, 3) symptoms observed, 4) test results to diagnose infectious diseases, and 5) vaccination or medication consumption (if relevant to the study question).
- **Smartphone sensor data** - consists of location, Wi-Fi, Bluetooth, screen, and activity.
- **Smartphone data** - consists of heart rate data, accelerometer, and gyroscope information and measures based on these data including active minutes, steps, distance, calories, and sleep level classification, including light, deep, REM, and awake periods.

Raw accelerometer data, mobile activity, and GPS locations are generally considered sensitive information. Following the data minimization principle, we did not extract this type of data for this research.

**References**

1. Oved, S. *et al.* Differential effects of COVID-19 lockdowns on well-being: Interaction between age, gender and chronotype. *J. R. Soc. Interface* **18**, (2021).

2. Gepner, Y. *et al.* Utilizing wearable sensors for continuous and highly-sensitive monitoring of reactions to the BNT162b2 mRNA COVID-19 vaccine. *Commun. Med.* **2**, (2022).

3. Margolis, K. L. *et al.* Frequency of adverse reactions after influenza vaccination. *Am. J. Med.* **88**, (1990).

4. Mofaz, M. *et al.* Self-Reported and Physiologic Reactions to Third BNT162b2 mRNA COVID-19 (Booster) Vaccine Dose. *Emerg. Infect. Dis.* **28**, (2022).

5. VÍVOSMART ® 4 Owner’s Manual. 2018. https://www8.garmin. com/manuals/webhelp/vivosmart4/EN-US/vivosmart_4_OM_ENUS. pdf (accessed June 10, 2023).

6. What Is the Stress Level Feature on My Garmin Watch? | Garmin Support. https://support.garmin.com/en-US/?faq=WT9BmhjacO4ZpxbCc0EKn9.

7. Pereira, T., Almeida, P. R., Cunha, J. P. S. & Aguiar, A. Heart rate variability metrics for fine-grained stress level assessment. *Comput. Methods Programs Biomed.* **148**, (2017).

8. Kim, H. G., Cheon, E. J., Bai, D. S., Lee, Y. H. & Koo, B. H. Stress and heart rate variability: A meta-analysis and review of the literature. *Psychiatry Investigation* vol. 15 (2018).

**Supplementary Figures**

**
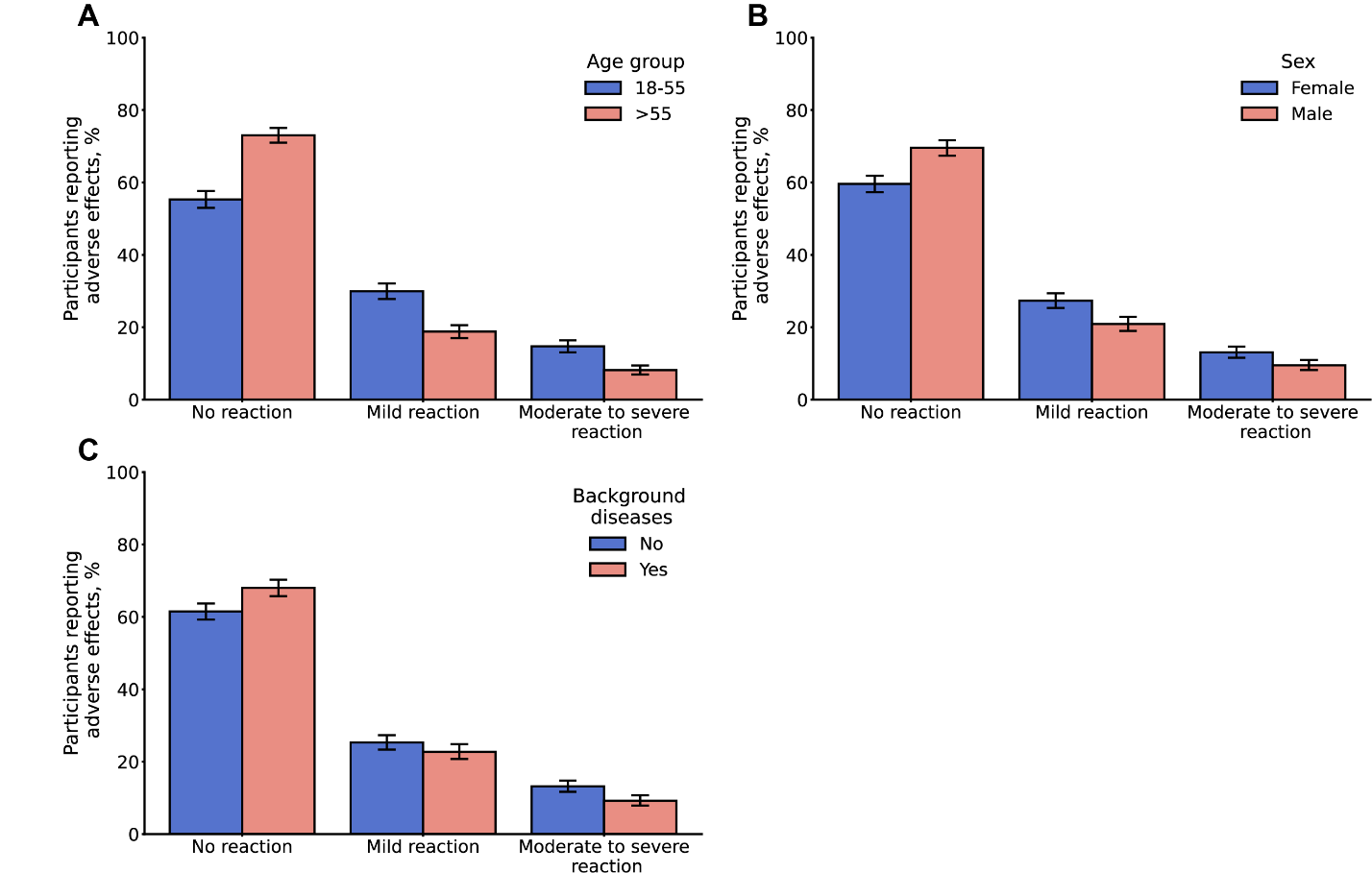
**

**Figure S1. Reaction severity following COVID-19 and/or Influenza vaccine by participant characteristics.** Percentage of all participants classified into each severity tier based on their most severe reported symptom in the 7 days following vaccination, stratified by: (**A**) Age, (**B**) Sex, and (**C**) Background diseases. Error bars represent 95% confidence intervals.


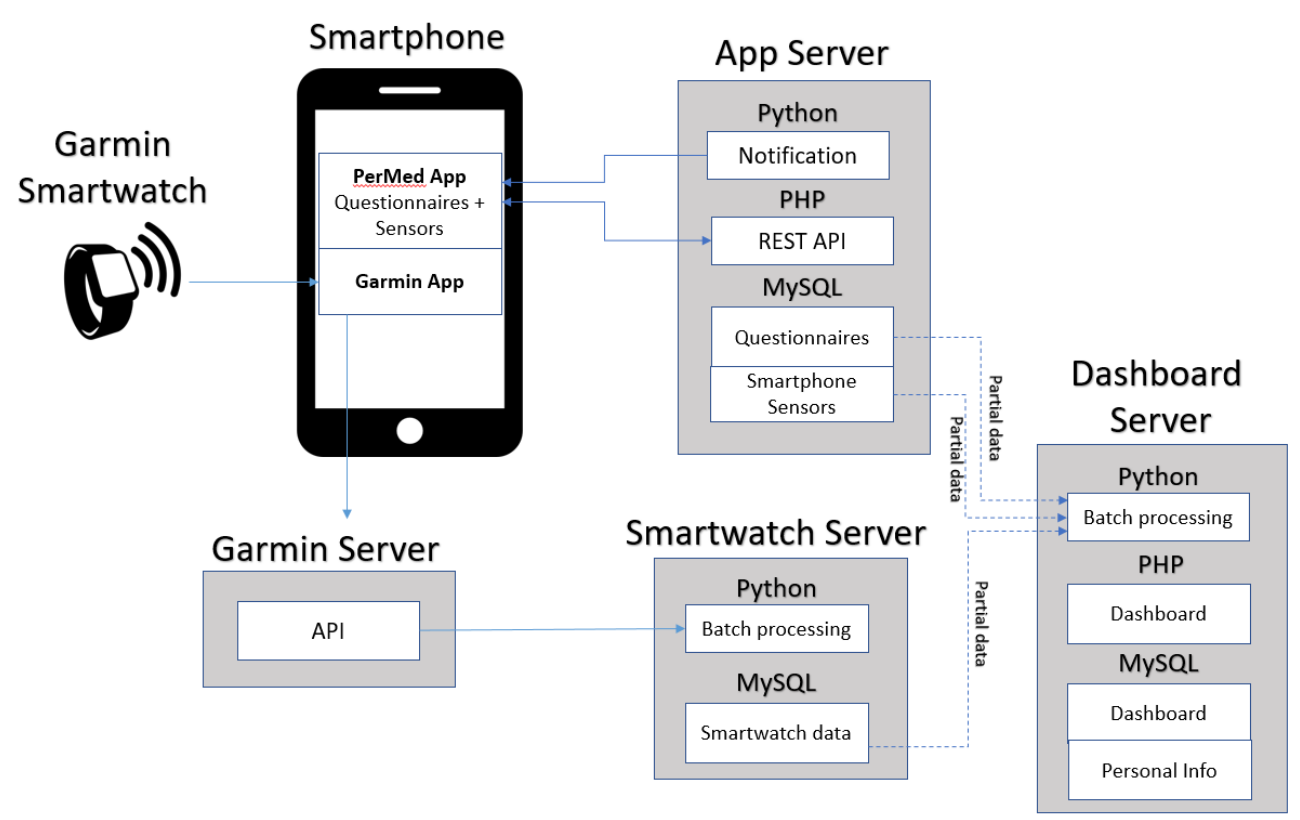


Figure S2. The high-level architecture of PerMed’s data collection platform.

**Supplementary Tables**

Table S1. Machine learning performance and hyperparameters for each machine learning method as well as for prediction and detection models.

| **Target** | **Model Type** | **AUROC±SE** | **Sensitivity±SE** | **Specificity±SE** | **Param** |
| --- | --- | --- | --- | --- | --- |
| Prediction | XGBClassifier | 0.69 ±0.05 | 0.72 ±0.07 | 0.64 ±0.11 | learning_rate: 0.1, max_depth: 3, min_child_weight: 4, n_estimators: 50 |
| Detection | XGBClassifier | 0.74 ± 0.03 | 0.75 ± 0.05 | 0.66 ± 0.09 | learning_rate: 0.1, max_depth: 3, min_child_weight: 3, n_estimators: 100 |
| Prediction | RF | 0.70 ±0.06 | 0.71 ±0.07 | 0.66 ±0.10 | max_depth: 3, min_samples_leaf: 1, min_samples_split: 10, n_estimators: 150 |
| Detection | RF | 0.74 ± 0.03 | 0.76 ± 0.05 | 0.64 ± 0.06 | max_depth: 6, min_samples_leaf: 4, min_samples_split: 10, n_estimators: 50 |
| Prediction | MLP | 0.69 ±0.06 | 0.80 ±0.05 | 0.56 ±0.10 | activation: tanh, hidden_layer_sizes: (100, 100), learning_rate: constant, solver: sgd} |
| Detection | MLP | 0.70 ± 0.06 | 0.73 ± 0.07 | 0.64 ± 0.10 | activation: tanh, hidden_layer_sizes: (100, 100), learning_rate: constant, solver: sgd |
| Prediction | KNN | 0.65 ±0.04 | 0.69 ±0.06 | 0.58 ±0.13 | n_neighbors: 15, weights: uniform |
| Detection | KNN | 0.68 ± 0.03 | 0.68 ± 0.07 | 0.64 ± 0.12 | n_neighbors: 15, weights: uniform |
